# The impact of recreational cannabis use on neuropsychological function in epilepsy

**DOI:** 10.1016/j.ebr.2023.100630

**Published:** 2023-10-26

**Authors:** Lucy Roberts-West, Sallie Baxendale

**Affiliations:** a University College Hospital, London, United Kingdom; b UCL Queen Square Institute of Neurology, Department of Clinical and Experimental Epilepsy, United Kingdom

**Keywords:** Cannabis, Epilepsy, Memory, Attention, Cognitive function

## Abstract

Cannabis use is associated with neuropsychological impairments in the general population, but little is known about the impact on cognitive function in people with epilepsy who are already at increased risk of difficulties due to the essential comorbidities of the disease. We compared the performance of 42 people with epilepsy (PWE) who reported regular cannabis use with 254 age matched, non-cannabis-using PWE. Patients completed tests of intellectual reserve, memory, language and processing speed. Approximately one in 17 patients (5.9 %) reported current cannabis use. Cannabis use was not associated with epilepsy type. Males were 1.8 times more likely to report cannabis use compared to females. Cannabis use was associated with lower intellectual reserve (Reading IQ: t = 2.8, p < 0.01, Cohen’s d = 0.49), reduced encoding of new information (List Learning: t = 3.3, p < 0.001, Cohen’s d = 0.56) and enhanced susceptibility to distraction on a subsequent recall task (t = 3.07, p < 0.01, Cohen’s d = 0.51. In regression models cannabis use was significantly associated with impairments in learning and recall after controlling for elevated levels of anxiety and depression. Our data indicates that recreational cannabis use in people with epilepsy amplifies deficits in new learning and enhances susceptibility to distraction in the retention of newly learnt material. Recreational cannabis use should be considered when interpreting the significance of these cognitive impairments when they are recorded in a clinical assessment.

## Highlights

• Recreational cannabis use is reported more commonly in males than females.
• Cannabis use is associated with lower baseline intellectual reserves.
• Cannabis use in people with epilepsy amplifies deficits in new learning.
• Enhanced susceptibility of recall to distraction is also evident in this group.

## Introduction

Cannabis is the most widely used recreational drug in the world and tops the table of ‘the most used drug’ in countries across the five continents [1]. However, whilst widespread, only eight countries have legalised its use for recreational purposes (Canada, Georgia, Luxembourg, Malta, Mexico, South Africa, Thailand, and Uruguay). Recreational use has also been legalised in some states in the USA. Other legislative approaches to the drug have been to decriminalise possession and users are not actively pursued by criminal law enforcement. Whilst it is still illegal to possess and use cannabis, civil penalties rather than criminal convictions will apply. This approach is more common in Europe, with Germany, Italy, Netherlands, Spain, Portugal and Switzerland adopting this approach.

Cannabis is classified as a class B drug in the UK. Penalties for possession include up to 5 years in prison, an unlimited fine or both [2], Nevertheless, cannabis is the most widely available and commonly used recreational drug in the UK with 7.6 % of adults reporting that they regularly or sometimes use the drug [3].

The possible anticonvulsant properties of cannabis have long been recognised [4]. Whilst some cannabis-based treatments were legalised in the UK in November 2018, very few NHS centres prescribe medicinal cannabis due to the limited and low-quality evidence of its efficacy to treat seizures in adults [5], [6] Thus, some people with epilepsy report using cannabis for ‘self-medicating’ purposes, in addition to recreational use[7]. The prevalence of cannabis use in adult epilepsy populations varies according to country, ranging from 6 % in Germany to 75 % in Canada [8], [9]. See Table 1.[8], [9], [10], [11], [12], [13], [14], [15], [16], [17], [18], [19].

**Table 1:** Self-Reported Recreational Cannabis Use in Epilepsy Populations.

Study	Country	Prevalence Rate	N Participants	Additional Findings
Feeney (1976)	USA (New Mexico)	<1% for adults over the age of 30 29 % for adults less than 30 years old	98	Most cannabis users reported no effect on seizures.
Gross et al. (2004)	Canada	21 % were active cannabis users: 13 % frequent users and 8 % heavy users	136	48 % had used cannabis in their lifetime. Most active users reported beneficial effects on seizures. In the regression analysis seizure frequency, longer disease duration and other illicit drug use were predictors of frequent cannabis use.
Saha et al. (2006)	South Africa	24 %	101	
Hamerle et al. (2014)	Germany	20 % had used cannabis since epilepsy diagnosis: 4 % were frequent active users	310	Current cannabis use was predicted by younger age and being male.
Massot-Tarrús & McLachlan (2016)	Canada	57 %	190	84 % of cannabis users perceived improvement in seizures. 85 % reported improvement in stress. 77 % reported improvement in sleep. 32 % of cannabis users perceived improvement in memory and concentration. 51 % report unchanged cognition. 17 % report worsened memory and concentration.
Suraev et al. (2017)	Australia	15 %	587	Cannabis use was predicted by the number of past AEDs tried, those who had a neurological condition in addition to epilepsy, the presence of pain, and epilepsy due to a structural brain abnormality. Most cannabis products were from illegal supplies with no awareness of its formal composition.
Moores et al. (2018)	Canada	30 %	140	25 % of participants had consumed cannabis in the past. Cannabis use was associated with increased seizure frequency. Side effects were common, the most frequent being mood.
Taalab et al. (2019)	Egypt	38 %	440	Most cannabis users reported that cannabis had a positive impact on their epilepsy condition.
von Wrede et al. (2019)	Germany	6 %	275	28 % reported past cannabis use.
Wahby et al. (2019)	Canada	21 %	337	Cannabis use independently associated with poor psychosocial health including depression, lower quality of life, worse epilepsy-related disability, and lower satisfaction with antiepileptic medication. Cannabis use partially mediated the negative effect of a psychiatric history on psychosocial outcomes.
Jackson-Tarlton et al. (2020)	Canada	19 %	43	Daily cannabis use was common, but there was no significant difference in use depending on diagnosis (newly diagnosed epilepsy, new-onset epilepsy, first unprovoked seizure).
Puteikis & Mameniškienė (2020)	Lithuania	16 %	250	Cannabis use was positively predicted by the belief that cannabis is safer because of its natural origin, and the premise of understanding its legal status. These patients were also more likely to consult internet sources and believe that cannabis is an effective epilepsy treatment option with no side effects.
Esmonde-White et al. (2023)	Canada	75 %: 70 % use cannabis at least once daily	395	221/395 participants had used cannabis within the past year Main reported side effects of cannabis use included impaired thinking (17 %), anxiety (16 %) and altered hunger (15 %). 60 % found cannabis was ‘somewhat’ to ‘very’ effective in reduce seizure frequency. Cannabis users main concerns about use included financial strain (37 %), lack of recommendation from a doctor (30 %) and a lack of information around cannabis use (19 %).

These widely disparate percentages are likely to reflect a number of factors including the legal status of the drug in the country, and associated ease of availability. Methodological differences in recording cannabis use will also impact reported rates (for example some studies ask about regular use, whilst others ask about use within the past year). There are also likely to be cultural differences in the rate of reporting what is an illegal activity for many, in a clinical or research setting.

A recent review by Li et al. (2023) [20] presents a comprehensive overview of ‘non-medical cannabis’ use in people with epilepsy. In their review of the literature, the median number of adults and children with epilepsy using cannabis was 24.5 %. Factors associated with non-medical cannabis use included male sex, younger adult age and lower education status, with cannabis use contributing to stigma and higher levels of depression. The reviewers suggest the evidence-base is sparse and heterogeneous with mostly small cross-sectional studies, and there are significant gaps in the literature including determining the neuropsychological effects of recreational cannabis use in people with epilepsy.

In non-patient populations, chronic cannabis use can lead to cognitive impairment including detrimental effects on learning, memory, executive function, global cognition, attention, and decision-making [21], [22], [23], [24], [25], [26], [27]. The most commonly reported cognitive impact is in the memory domain with encoding, storage, manipulation, and retrieval of verbal information affected [28], [29], [30]. Some studies report neutral or mildly beneficial effects of medicinal cannabidiol on neuropsychological function in people with epilepsy [31], [32], [33] which may be secondary to improved seizure control. These inconsistent findings may reflect, at least in some part, the significant methodological challenges of studies in this field. In non-experimental settings it is difficult to control for the heterogeneity of recreational cannabis use with respect to duration and frequency of use, potency, age of onset and periods of abstinence [25], [30], [34], [35], [36].

THC is the psychoactive component of cannabis acting through the endocannabinoid system which regulates mood, learning, and memory [37] and has a high content within current strains of cannabis in the UK [38]. Strains with higher Δ^9^-tetrahydrocannabinol (THC) content are thought to result in greater cognitive impairment and mood disturbance [39].

Little is known about the additive impact of recreational cannabis use on neuropsychological function in adults with epilepsy, with this population already at-risk of memory and cognitive difficulties due to the essential comorbidities of the condition. The current study aimed to examine the impact of cannabis use on cognitive function in this group.

## Methods

The records of a consecutive series of 800 patients who underwent a neuropsychological assessment in the epilepsy service at the University College London Hospital between 2019 and 2022 were examined for references to recreational cannabis use. All patients who attend for a neuropsychological assessment in our department are routinely asked about recreational drug use in their clinical interview, prior to their formal assessment with standardised tests. Information regarding recreational drug use is also recorded in the medical and neuropsychiatric assessments they undergo when assessed by the multidisciplinary team. We identified seventy patients who admitted using cannabis for recreational purposes, either in the past or at the time of the assessment, comprising 8.75 % of our referrals. Forty-seven of these patients reported current use of cannabis.

Three of the patients who reported current cannabis use had an exclusive diagnosis of non-epileptic attack disorder and were excluded from the analyses of cognitive performance. Two patients had undergone surgery prior to the assessment and were also excluded from these analyses to avoid the confound of the impact of surgery on cognitive performance. Following these exclusions, our search strategy identified 42 patients (27 males, 15 females) with epilepsy who reported current recreational cannabis use at the time of their neuropsychological assessment.

### Comparator group

The neuropsychological test scores of the cannabis group were compared to those of a consecutive series of 254 (144 males, 110 females) age-matched, non cannabis using people with epilepsy who underwent an assessment over the same time period. Since none of the cannabis group had an intellectual disability, patients with an intellectual disability were excluded from the comparator group. Intellectual disability was established as part of the clinical neuropsychological assessment which consisted of medical and educational review and the completion of formal tests of cognitive function. As with the cannabis group, patients who were referred for a postoperative assessment were excluded.

### Neuropsychological measures

The neuropsychological assessment included a standardised measure of pre-morbid ability, the National Adult Reading Test [40] and tests of intellectual, memory, language and executive function, in addition to questionnaire data. The scaled scores from the Digit Span and Coding tests from the Wechsler Adult Intelligence Scale (WAIS-IV)[41] were used as measures of working memory and processing speed respectively. Measures of memory included the List 1–5 score from the BMIPB II [42] list learning task (a measure of learning over 5 trials) and the List A6 score (a measure of retention following distraction). The design learning score from the BMIPB was used as our measure of visual memory. The total number of items correctly named on the Graded Naming Test was used as a measure of language function. The number of words generated in 60 s beginning with ‘S’ and number of animals generated were our measures of phonetic and sematic fluency. Self-report measures included the Hospital Anxiety and Depression Scale (HADS) [43] and the Subjective Memory Questionnaire (SMQ). All of these measures have been described in detail previously[44].

The tests that comprise a clinical neuropsychological assessment are determined on an individual basis and are selected in response to the specific referral received. Not all patients undertake all tasks. This is reflected in the numbers in each analyses.

### Ethical Approval

All data was fully anonymised prior to the analyses in order to conform to ethical approvals granted for the analysis of routinely collected clinical data in an audit of recreational cannabis use in patients referred for a neuropsychological assessment. (Hospital Board Approval: 20–202223-SE).

### Data availability statement

The clinical data analysed in the current study are not publicly available due to patient privacy and restricted access, but further information about the database is available from the corresponding author on reasonable request.

### Statistical analyses

All analyses were conducted using SPSS v27. Differences between the cannabis group (n = 42) and the comparator group on the neuropsychological measures were explored with independent t tests. Linear regression models were used to examine the relative contributions of cannabis status and mood to measures of cognitive function, where preliminary analyses indicated significant group differences in function.

## Results

### Demographic & clinical characteristics of cannabis users

8.75 % of the consecutive series of 800 patients who attended our department for a neuropsychological assessment reported past or present recreational cannabis use. Approximately one in 17 patients (5.9 %) reported current cannabis use at the time of the assessment (n = 47). In the consecutive series of 800 patients, cannabis use (current/past/never) was not associated with epilepsy type (Focal, Generalised, Unclassified, Non Epileptic Attack Disorder, Dual Diagnosis) Chi square = 14.9, df 8, p > 0.05.

Typically more females are referred for an assessment in our department than males (56.3 %). However, in our comparator group, which was age-matched to the cannabis group and which excluded surgical patients, males represented 56 % of the group. In the cannabis group, males represented 64 % of the group (chi square 0.8, df 1, p > 0.05).

Males were approximately 1.8 times more likely to use cannabis compared to females although this just failed to reach statistical significance (Males n = 27; Females n = 15, χ^2^ = 3.4, df 1, p = 0.06). Patients in our series who reported current cannabis use were also significantly younger than past and non-cannabis users (f (2,521) = 8.4, p < 0.001) with a small-moderate effect size (η2 = 03).

### Neuropsychological test scores

The clinical and demographic characteristics of the cannabis group and the age matched comparator group are presented in Table 2. Cannabis use was associated with lower intellectual reserve (NART IQ) reduced verbal learning (List Learning) and enhanced susceptibility to distraction on a subsequent recall task (List A6). The cannabis group also reported significantly higher levels of anxiety and depression on the HADS. No significant differences between the cannabis group and the comparator group were evident on tests of working memory (Digit Span), processing speed (Coding), immediate or delayed prose recall, visual learning, naming or verbal fluency scores. See Table 3.

**Table 2:** Demographic and clinical characteristics of the sample.

	Epilepsy Group (n = 42)	Control Group (n = 254)
Age	30.38 (9.2)	30.95 (9.7)
Sex	27 males, 15 females	144 males, 110 females
Epilepsy Type	Focal n = 31 Generalised n = 6 Unclassified n = 2 Dual diagnosis n = 3	Focal n = 160 Generalised n = 36 Unclassified n = 45 Dual diagnosis n = 13

**Table 3:** Neuropsychological Test Scores in the Cannabis and Control Groups.

Variable	Group	N*	Mean (std Dev)	Z score**	Levene's F	Levene's Sig.	t-value	df	Sig.	Cohen's d
Reading IQ	Non cannabis	189	99.60 (8.97)	−0.02	1.10	0.30	2.82	226	0.003	0.49
	Cannabis	39	94.97 (10.95)	−0.33						
Digit Span	Non cannabis	215	7.83 (3.8)	−1.44	0.02	0.88	−0.75	251	0.23	−0.13
	Cannabis	38	8.24 (2.98)	−1.17						
Processing speed (Coding)	Non cannabis	220	7.68 (3.1)	−1.54	3.92	0.05	1.29	253	0.10	0.23
	Cannabis	35	6.91 (2.93)	−2.06						
Verbal Learning (list)	Non cannabis	245	45.32 (11.84)	−0.82	0.48	0.49	3.36	285	<0.001	0.56
	Cannabis	42	38.76 (10.77)	−1.45						
Verbal recall (after distraction)	Non cannabis	245	9.18 (3.62)	−1.7	2.86	0.09	3.07	285	0.001	0.51
	Cannabis	42	7.36 (3.04)	−2.5						
Story (immediate recall)	Non cannabis	183	18.79 (9.46)	−1.18	0.44	0.51	1.28	203	0.10	0.29
	Cannabis	22	16.09 (8.17)	−1.46						
Story (delayed recall)	Non cannabis	181	16.67 (10.78)	−1.19	1.17	0.28	1.20	201	0.12	0.27
	Cannabis	22	13.82 (7.75)	−1.49						
Visual Learning	Non cannabis	227	36.39 (10.77)	−0.16	5.69	0.02	1.17	265	0.12	0.20
	Cannabis	40	34.30 (8.27)	−0.36						
Graded Naming	Non cannabis	209	14.06 (5.03)	−1.54	0.76	0.38	−0.74	234	0.23	−0.15
	Cannabis	27	14.81 (4.77)	−1.36						
Fluency (semantic)	Non cannabis	228	18.74 (6.43)	−0.25	0.02	0.89	0.98	266	0.16	0.17
	Cannabis	40	17.65 (6.76)	−0.51						
Fluency (phonetic)	Non cannabis	225	12.16 (5.58)	−0.83	1.70	0.19	−0.84	263	0.20	−0.14
	Cannabis	40	12.98 (6.25)	−0.77						
HADS Anxiety	Non cannabis	202	9.60 (4.55)	–	3.74	0.05	−2.62	238	0.01	−0.46
	Cannabis	38	11.66 (3.74)	–						
HADS Depression	Non cannabis	202	5.87 (4.19)	–	0.06	0.80	−3.32	238	<0.001	−0.59
	Cannabis	38	8.34 (4.31)	–						
Subjective Memory Score	Non cannabis	195	61.56 (19.96)	–	0.24	0.63	−1.46	227	0.07	−0.27
	Cannabis	40	56.15 21.75)	–						

* Not all patients undertake all tasks. This is reflected in the numbers in each analyses.

Shaded rows indicate significant group differences.

** z scores based on the healthy population mean. These are not available for the questionnaire measures.

Since elevated levels of anxiety and low mood can contribute to poor scores on neuropsychological tests we examined the relative contributions of cannabis use and the HADS Anxiety and HADS depression scores to the List learning and List Recall scores via linear regression.

### List learning: Regression analyses

A univariate linear regression analysis was conducted to examine the relationship between verbal learning as the dependent variable (List Learning A1-5) and the independent variables: “HADS anxiety score,” “HADS depression Score”, “premorbid level of function” (NART IQ) and “cannabis use group” (Yes/No).

The results of the analysis are presented in Table 4. The corrected model, which includes the independent variables “HADS Anxiety,” “HADS Depression,” “NART IQ,” and “Cannabis Group,” was found to be significant, F(4, 198) = 7.992, p < 0.001. This indicates that the model as a whole had a significant effect on the dependent variable “List 1–5.” Specifically, “Nart IQ” (F = 21.455, p < 0.001) and “Cannabis Group)” (F = 4.326, p = 0.039) made significant contributions to the model, while “HADS Anxiety” (F = 0.049, p = 0.824) and “HADS Depression” (F = 0.340, p = 0.560) were not significant predictors. The overall model explained a small, but significant proportion of the variance in “List 1-5″ (R^2^ = 0.139, Adjusted R^2^ = 0.122).

**Table 4:** Linear Regression Results; Tests of Between-Subjects Effects List Learning (List A1-5).

	Type III Sum of Squares	df	Mean Square	F	Sig.
Corrected Model	3301.455a	4	825.364	7.992	<0.001
Intercept	70.312	1	70.312	0.681	0.41
HADS Anxiety	5.111	1	5.111	0.049	0.824
HADS Depression	35.136	1	35.136	0.34	0.56
NART IQ	2215.628	1	2215.628	21.455	<0.001
Cannabis Group	446.713	1	446.713	4.326	0.039
Error	20447.235	198	103.269		
Total	443,691	203			
Corrected Total	23748.69	202			

### Recall following distraction: Regression analyses

A second univariate linear regression analysis was performed to investigate the relationship between the dependent variable “List A6″ and the independent variables ”HADS Anxiety,“ ”HADS Depression,“ ”NART IQ,“ and cannabis use group (Yes/No). The results of the analysis are presented in Table 5. The corrected model, including the independent variables ”HADS Anxiety,“ ”HADS Depression,“ ”NART IQ,“ and ”Cannabis Group,“ showed statistical significance, F(4, 198) = 5.667, p < 0.001. This suggests that the model as a whole had a significant impact on the dependent variable ”List A6.“ As with the predictors of verbal learning (List 1–5) ”NART IQ“ (F = 11.934, p < 0.001) and ”Cannabis group“ (F = 5.316, p = 0.022) made statistically significant contributions to the model, whereas self-reported mood scores on the HADS did not: ”HADS Anxiety“ (F = 0.012, p = 0.912); ”HADS Depression“ (F = 0.294, p = 0.588). Whilst the overall model accounted for a statistically significant portion of the variance in ”List A6″ (R^2^ = 0.103, Adjusted R^2^ = 0.085), the portion was small.

**Table 5:** Linear Regression Results; Tests of Between-Subjects Effects Recall following distraction (LIST A6).

	Type III Sum of Squares	df	Mean Square	F	Sig.
Corrected Model	232.475a	4	58.119	5.667	<0.001
Intercept	0.093	1	0.093	0.009	0.924
HADS Anxiety	0.125	1	0.125	0.012	0.912
HADS Depression	3.014	1	3.014	0.294	0.588
NART IQ	122.393	1	122.393	11.934	<0.001
Cannabis Group	54.52	1	54.52	5.316	0.022
Error	2030.589	198	10.256		
Total	19,841	203			
Corrected Total	2263.064	202			

## Discussion

In our tertiary referral centre, 8.75 % of patients referred for a neuropsychological assessment reported past or present cannabis use, with 5.9 % reporting current use. Cannabis use was more commonly reported by males than females and the cannabis users were typically younger than the wider cohort. The cannabis group displayed lower intellectual reserve, reduced verbal learning abilities, and increased susceptibility to distraction on a verbal recall task compared to the matched comparator group, in a pattern consistent with that reported in a recent comprehensive review of cannabis use in this population [20]. The patients in the cannabis group reported higher levels of anxiety and depression on the HADS. Linear regression analyses revealed that whilst premorbid level of intellectual function was a significant predictor of performance, cannabis use was also independently associated with poorer verbal learning and recall following distraction, even after controlling for elevated levels of anxiety and depression.

Our results are consistent with previous studies that have shown associations between cannabis use and cognitive impairments in healthy individuals, particularly in the verbal learning and memory domains [25], [28], [30]. Whilst several studies have reported that cannabis use, particularly heavy and long-term use, is associated with deficits in various cognitive domains, including attention, memory, executive function, and processing speed [21], [22], [23], [24], [26], [45] the deficits recorded in our sample were primarily limited to the verbal memory and attention domains. The increased susceptibility to distraction on a recall task among cannabis users aligns with research suggesting that cannabis use can impair attentional processes and is associated with difficulties in maintaining attention and inhibiting distractions [22], [28]. However it is also possible that poor performance on recall following distraction was a hangover from the original, shallow encoding across the five trials of the list learning task in out sample. We did not have the final trial score disaggregated from the total list learning score in this dataset available to test this hypothesis further. Further work is underway to examine the relationship between the final trial score on the list learning task and the subsequent recall score in the people with epilepsy and we plan to include cannabis use as a variable of interest in this wider analysis.

It is interesting that it is the language dominant memory networks that appear to be primarily impacted by cannabis use in our sample. We have observed a similar pattern when we have looked at the exacerbatory impacts of anxiety and low mood on neuropsychological test performance in people with epilepsy [46]. These functions appear to be particularly vulnerable to factors that impact on the cognitive reserves of people with epilepsy, which may already be compromised due to organic factors associated with seizures and the impacts of anti-seizure medications. However, whilst this pattern is seen in relation to other factors that impact cognitive function, it is unclear why language dominant networks would be primarily affected. It may reflect something specific to the language dominant networks themselves or may be an artefact of our test construction. Our tests of verbal ability may tap into more discreet networks, whilst performance on tests of non-verbal ability may be more widely distributed, allowing for more pressure on the system before a deficit becomes evident. Insights from functional imaging paradigms may be able to elucidate this further.

There are many reasons why people with epilepsy experience difficulties in multiple cognitive domains, including the underlying pathology responsible for their seizures, the effects of seizures and sub-clinical EEG abnormalities, the impacts of antiseizure medications and common comorbidities associated with the condition [47], [48]. The fact that we did not find impacts of cannabis use on a wider range of cognitive functions, as would be predicted by the literature in healthy individuals may reflect the fact that these functions are already often compromised by epilepsy related variables [49]. It is also worth noting that the cognitive impairments associated with cannabis use can be subtle and may not be evident in all individuals or under all conditions [50]. Factors such as age, duration and frequency of use, potency of cannabis, and individual differences in vulnerability are likely to influence the magnitude and persistence of cognitive deficits.

The elevated levels of anxiety and depression in our cannabis group is consistent with previous research linking cannabis use to increased psychological distress [51]. Self medicating with cannabis for anxiety and low mood is often reported as a primary motivation by people who use the drug on a recreational basis [52]. Our study design does not allow for casual inferences and we can only note the increased association of cannabis use in our group in young men with limited cognitive reserve. It is well established that men are less likely to access mental health services as readily as women, with stigma and help-seeking attitudes identified as key facets contributing to males seeking less support for mental health difficulties [53], [54]. In the UK, a disproportionately low number of males compared to females are referred to mental health services and receive mental health treatments [55]. As such, a tendency for males to self-medicate with alcohol and drugs to ease emotional distress has been recognised [53], [54] which may explain the association between males and ‘self-medicating’ cannabis use within the present study. Additional barriers to accessing mental services are associated with limited cognitive reserve. In addition to cognitive factors, other barriers to accessing mental health services may include long waiting lists and inadequate provision resulting in individuals turning to alternative remedies for their difficulties.

However it is also possible that cannabis use has a bidirectional relationship with lower cognitive reserve. Whilst those with more limited cognitive reserve may be more likely to self-medicate due to the barriers they face accessing mental health services, cannabis use may also result in more limited cognitive reserves, particularly if people began using it during their adolescence, when neurodevelopment was still underway. Longitudinal studies have shown that cannabis use during adolescence and young adulthood, when the brain is still developing, may have a more pronounced negative impact on cognitive abilities [27]. Persistent cannabis use in adolescence or young adulthood may interfere with critical brain development processes resulting in global blunting of neuropsychological function, as reported by Meier et al. (2012) [27]. Due to the existing vulnerability of brain function in people with epilepsy, the additional neuropsychological burden of cannabis use during young adulthood may have negative impacts on the development of a broad range of neuropsychological functions. This could possibly account for our finding that the cannabis users within this study had significantly lower reading IQ than non-cannabis users. Further data establishing the age of onset of cannabis use will be needed to investigate these possibilities.

### Limitations

There are several limitations of the study. The data relied on self-report measures of recreational cannabis use. Given that cannabis use remains illegal in the UK, patient reports are likely to underestimate true cannabis use in this sample. Moreover, the sample consisted of patients who were referred for a clinical assessment of their cognitive function. Whilst the reasons for their referral varied, all patients will have been seen due to concerns about cognition, arising from the patient, their family or the treating clinician or in the context of a presurgical assessment for medically intractable epilepsy. These patients are not representative of the majority of people with epilepsy whose seizures are well controlled on medication and who are never referred for a formal neuropsychological assessment. The pragmatic sample and observational design limit our ability to establish causal relationships or assess the long-term or cumulative effects of cannabis use. Caution should therefore be used in generalising these findings to the broader population.

### Future research

Whilst the legal status of recreational cannabis currently prohibits any kind of controlled trial to investigate the impact of recreational, non-prescribed use on cognitive function in many countries, such research may be possible in jurisdictions where the use of cannabis has been legalised, although ethical considerations may prevent any study designs involving randomisation, even in these settings. In other regions where recreational cannabis remains proscribed, follow-up assessments tracking the cognitive performance of those who report cannabis use to examine potential recovery or reversibility of cognitive deficits with cessation, would provide valuable insights to share with this patient population. Whilst we did not find any association between epilepsy type and cannabis use, further investigations into the interactions between cannabis use and different anti-seizure medications and impact on cognitive function may be a useful area of research. In a larger sample, the exploration of potential dose–response relationships and the impact of different cannabis strains and modes of consumption could also further our understanding of the cognitive effects of cannabis in this patient group.

## Conclusions

In summary, we found that approximately one in 17 of the patients referred to our service reported recreational cannabis use. Usage was most commonly reported by young males with elevated levels of anxiety and depression. Recreational cannabis use was associated with lower intellectual reserve, reduced verbal learning, and increased susceptibility to distraction on a recall task. These effects remained significant even after controlling for elevated levels of anxiety and depression. The findings underscore the importance of considering cannabis use as a potential factor influencing cognitive function in neuropsychological assessments, particularly in presurgical settings where deficits in verbal memory may be misattributed.

## Ethical statement

This study examined patient data collected on a routine clinical basis. All data was fully anonymised prior to the analyses in order to conform to the ethical approvals granted for the analysis of this data an audit of recreational cannabis use in patients referred for a neuropsychological assessment. (Hospital Board Approval: 20–202223-SE). No individuals can be identified from the information presented in this study. The clinical data analysed in the current study are not publicly available due to patient privacy and restricted access, but further information about the database is available from the corresponding author on reasonable request.

## Declaration of Competing Interest

The authors declare that they have no known competing financial interests or personal relationships that could have appeared to influence the work reported in this paper.

## Data Availability

Data will be made available on request.

## References

[1] World Drug Report 2021. Mapped: The most common illicit drugs in the world. 2021 n.d. https://www.visualcapitalist.com/mapped-the-most-common-illicit-drugs-in-the-world/.

[2] UK Government. Drugs Penalties 2023.

[3] Home Office National Statistics. Drug Misuse: Findings from the 2018/19 crime survey for England and Wales 2019.

[4] Russo E.B. (2017). Cannabis and epilepsy: an ancient treatment returns to the fore. Epilepsy Behav.

[5] Kirkpatrick M., O’callaghan F. (2022). Epilepsy and cannabis: so near, yet so far. Dev Med Child Neurol.

[6] Schlag AK, Zafar R, Nutt D. Medical cannabis and epilepsy in the UK – A qualitative analysis of the carers’ perspective: We’re asking for quality of life for our children. Drug Sci Policy Law 2021;7:205032452110349–205032452110349. doi:10.1177/20503245211034930.

[7] Kerr A., Walston V., Wong V.S.S., Kellogg M., Ernst L. (2019). Marijuana use among patients with epilepsy at a tertiary care center. Epilepsy Behav.

[8] Esmonde-White C., McLachlan R.S., Burneo J., Arts J., Redhead C., Suller M.A. (2023). Nationwide study of postlegalization marijuana use among patients with epilepsy in Canada. Neurol Clin Pract.

[9] von Wrede R., Moskau-Hartmann S., Amarell N., Elger C.E., Helmstaedter C. (2019). Knowledge, expectations and fears of cannabis use of epilepsy patients at a tertiary epilepsy center. Epilepsy Behav.

[10] Jackson-Tarlton C.S., Whatley B.P., Kasheke G.D.S., Pohlmann-Eden B., Omisade A. (2020). A prospective pilot study of cognitive impairment and mood in adults with first seizure, new-onset epilepsy, and newly diagnosed epilepsy at time of initial seizure presentation. Epilepsy Behav.

[11] Puteikis K., Mameniškienė R. (2020). Use of cannabis and its products among patients in a tertiary epilepsy center: a cross-sectional survey. Epilepsy Behav.

[12] Feeney D.M. (1976). Marihuana use among epileptics. JAMA.

[13] Gross D.W., Hamm J., Ashworth N.L., Quigley D. (2004). Marijuana use and epilepsy: prevalence in patients of a tertiary care epilepsy center. Neurology.

[14] Saha S.K., Nel M., Prinsloo E.A.M. (2006). Profile and associated factors for re-admitted epileptic patients with complications in a South African hospital. Cent Afr J Med.

[15] Massot-Tarrús A., McLachlan R.S. (2016). Marijuana use in adults admitted to a Canadian epilepsy monitoring unit. Epilepsy Behav.

[16] Suraev A.S., Todd L., Bowen M.T., Allsop D.J., McGregor I.S., Ireland C. (2017). An Australian nationwide survey on medicinal cannabis use for epilepsy: history of antiepileptic drug treatment predicts medicinal cannabis use. Epilepsy Behav.

[17] Moores G., Lockey A., Attar A. (2018). High times? prevalence and perceptions of marijuana use among patients with epilepsy. Neurology.

[18] Taalab Y.M., Fathi Mohammed W., Helmy M.A., Othman A.A.A., Darwish M., Hassan I. (2019). Cannabis influences the putative cytokines-related pathway of epilepsy among egyptian epileptic patients. Brain Sci.

[19] Wahby S., Karnik V., Brobbey A., Wiebe S., Sajobi T., Josephson C.B. (2019). Cannabis use is both independently associated with and mediates worse psychosocial health in patients with epilepsy. J Neurol Neurosurg Psychiatry.

[20] Li J., Areal C.C., Toffa D.H., Citherlet D., Deacon C., Jutras-Aswad D. (2023). Use of non-medical cannabis in epilepsy: a scoping review. Front Neurol.

[21] Burggren A.C., Shirazi A., Ginder N., London E.D. (2019). Cannabis effects on brain structure, function, and cognition: considerations for medical uses of cannabis and its derivatives. Am J Drug Alcohol Abuse.

[22] Figueiredo P.R., Tolomeo S., Steele J.D., Baldacchino A. (2020). Neurocognitive consequences of chronic cannabis use: a systematic review and meta-analysis. Neurosci Biobehav Rev.

[23] Ganzer F., Bröning S., Kraft S., Sack P.-M., Thomasius R. (2016). Weighing the evidence: a systematic review on long-term neurocognitive effects of cannabis use in abstinent adolescents and adults. Neuropsychol Rev.

[24] Gonzalez R., Pacheco-Colón I., Duperrouzel J.C., Hawes S.W. (2017). Does cannabis use cause declines in neuropsychological functioning? A review of longitudinal studies. J Int Neuropsychol Soc.

[25] Kroon E., Kuhns L., Cousijn J. (2021). The short-term and long-term effects of cannabis on cognition: recent advances in the field. Curr Opin Psychol.

[26] Lovell M.E., Akhurst J., Padgett C., Garry M.I., Matthews A. (2020). Cognitive outcomes associated with long-term, regular, recreational cannabis use in adults: a meta-analysis. Exp Clin Psychopharmacol.

[27] Meier M.H., Caspi A., Ambler A., Harrington H., Houts R., Keefe R.S.E. (2012). Persistent cannabis users show neuropsychological decline from childhood to midlife. PNAS.

[28] Broyd S.J., van Hell H.H., Beale C., Yücel M., Solowij N. (2016). Acute and chronic effects of cannabinoids on human cognition—a systematic review. Biol Psychiatry.

[29] Kroon E., Kuhns L., Hoch E., Cousijn J. (2020). Heavy cannabis use, dependence and the brain: a clinical perspective. Addiction.

[30] Solowij N., Battisti R. (2008). The chronic effects of cannabis on memory in humans: a review. Curr Drug Abuse Rev.

[31] Gaston T.E., Allendorfer J.B., Nair S., Bebin E.M., Grayson L.P., Martin R.C. (2020). Effects of highly purified cannabidiol (CBD) on fMRI of working memory in treatment-resistant epilepsy. Epilepsy Behav.

[32] Martin R.C., Gaston T.E., Thompson M., Ampah S.B., Cutter G., Bebin E.M. (2019). Cognitive functioning following long-term cannabidiol use in adults with treatment-resistant epilepsy. Epilepsy Behav.

[33] Metternich B., Wagner K., Geiger M.J., Hirsch M., Schulze-Bonhage A., Klotz K.A. (2021). Cognitive and behavioral effects of cannabidiol in patients with treatment-resistant epilepsy. Epilepsy Behav.

[34] Hirst R., Vaughn D., Arastu S., Deneen A., Pilavjian H. (2021). Female sex as a protective factor in the effects of chronic cannabis use on verbal learning and memory. J Int Neuropsychol Soc.

[35] Prini P., Zamberletti E., Manenti C., Gabaglio M., Parolaro D., Rubino T. (2020). Neurobiological mechanisms underlying cannabis-induced memory impairment. Eur Neuropsychopharmacol.

[36] Schoeler T., Bhattacharyya S. (2013). The effect of cannabis use on memory function: an update. Subst Abuse Rehabil.

[37] Rosenberg E.C., Tsien R.W., Whalley B.J., Devinsky O. (2015). Cannabinoids and epilepsy. Neurotherapeutics.

[38] Potter D.J., Hammond K., Tuffnell S., Walker C., Di Forti M. (2018). Potency of Δ ^9^ -tetrahydrocannabinol and other cannabinoids in cannabis in England in 2016: implications for public health and pharmacology. Drug Test Anal.

[39] Morgan C.J.A., Gardener C., Schafer G., Swan S., Demarchi C., Freeman T.P. (2012). Sub-chronic impact of cannabinoids in street cannabis on cognition, psychotic-like symptoms and psychological well-being. Psychol Med.

[40] Nelson HE, Willison J. National Adult Reading Test (NART) 1991.

[41] Wechsler D. Wechsler Adult Intelligence Scale--Fourth Edition (WAIS-IV) 2008.

[42] M O, Coughlan A, Crawford J. BIRT Memory & Information Processing Battery 2007.

[43] Zigmond A.S., Snaith R.P. (1983). The hospital anxiety and depression scale. Acta Psychiatr Scand.

[44] Baxendale S., Thompson P. (2020). The association of cognitive phenotypes with postoperative outcomes after epilepsy surgery in patients with temporal lobe epilepsy. Epilepsy Behav.

[45] Meier M.H., Caspi A., R. Knodt A., Hall W., Ambler A., Harrington H. (2022). Long-term cannabis use and cognitive reserves and hippocampal volume in midlife. Am J Psychiatry.

[46] Baxendale S.A. (2022).

[47] Baxendale S. The who, what, when and why of cognitive difficulties in epilepsy. Https://DoiOrg/1012968/Hmed20220534 2023:1–8. doi:10.12968/HMED.2022.0534. 10.12968/hmed.2022.0534 37127413

[48] Baxendale S., Thompson P. (2010). Beyond localization: the role of traditional neuropsychological tests in an age of imaging. Epilepsia.

[49] Wilson S.J., Baxendale S., Barr W., Hamed S., Langfitt J., Samson S. (2015). Indications and expectations for neuropsychological assessment in routine epilepsy care: report of the ILAE neuropsychology task force, diagnostic methods commission, 2013–2017. Epilepsia.

[50] Scott J.C., Slomiak S.T., Jones J.D., Rosen A.F.G., Moore T.M., Gur R.C. (2018). Association of cannabis with cognitive functioning in adolescents and young adults a systematic review and meta-analysis. JAMA Psychiatry.

[51] Rup J., Freeman T.P., Perlman C., Hammond D. (2021). Cannabis and mental health: prevalence of use and modes of cannabis administration by mental health status. Addict Behav.

[52] Wallis D., Coatsworth J.D., Mennis J., Riggs N.R., Zaharakis N., Russell M.A. (2022). Predicting self-medication with cannabis in young adults with hazardous cannabis use. Int J Environ Res Public Health.

[53] Bell O.J., Flynn D., Clifford T., West D., Stevenson E., Avery L. (2023). Identifying behavioural barriers and facilitators to engaging men in a community-based lifestyle intervention to improve physical and mental health and well-being. Int J Behav Nutr Phys Act.

[54] Sagar-Ouriaghli I, Godfrey E, Bridge L, Meade L, Brown JSL. Improving mental health service utilization among men: a systematic review and synthesis of behavior change techniques within interventions targeting help-seeking. Am J Mens Health 2019;13:155798831985700–155798831985700. doi:10.1177/1557988319857009. 10.1177/1557988319857009 PMC6560805 31184251

[55] McManus S., Bebbington P., Jenkins R., Brugha T. (2014). Mental health and wellbeing in England. Adult Psychiatric Morbidity Survey.

